# Supplementary material for: Isolation of Subtype 3c, 3e and 3f-Like Hepatitis E Virus Strains Stably Replicating to High Viral Loads in an Optimized Cell Culture System
Source: Viruses. 2019 May 28;11(6):483. doi: 10.3390/v11060483 (PMC6632007; doi:10.3390/v11060483)
Supplement: Supplementary file 1 [file viruses-11-00483-s001.zip › viruses-499710 final supplementary/Table S1.pdf]

Sequenced genome segments of subtype 3c strain 14-16753

Sequence Segment	Location (on 14-16753 genome, nested Primer excluded)	Primer<br>First round PCR	Nested PCR	Sequencing
5'n<br>$T_{A}f$ = 68 °C<br>$T_{A}n$ = 68 °C	-7 – 483	5'-RACE_s<br>5'-GS1_as<br>LUP<br>SUP	NUP<br>5'-GS1n_as	NUP<br>1_s<br>S_s<br>Sn_s<br>289_as<br>393_as<br>440_as<br>5'-GS1n_as
Sn<br>$T_{A}f$ = 58 °C<br>$T_{A}n$ = 58 °C	132 – 565	S_s<br>S_as	Sn_s<br>Sn_as	2_s<br>1_as<br>Sn_as
2n_12<br>$T_{A}f$ = 58 °C<br>$T_{A}n$ = 58 °C	473 – 1031	1_s<br>3_as_m1	2_s<br>2_as_m1	2_s<br>2_as_m1
A1<br>$T_{A}f$ = 58 °C<br>$T_{A}n$ = 58 °C	750 – 1198	A_s<br>A_as	An1_s<br>An1_as	An1_s<br>3_s<br>2_as_m1<br>An1_as
2n_7<br>$T_{A}f$ = 58 °C<br>$T_{A}n$ = 58 °C	999 – 2042	2_s<br>3200_as_m1	3_s<br>4_as_m1	3_s<br>4_s<br>1500s_m1<br>3_as_m1<br>1800_as_m1<br>4_as_m1
A2<br>$T_{A}f$ = 68 °C<br>$T_{A}n$ = 68 °C	1689 – 2117	A_s<br>Aas	An2_s<br>An2_as	An2_s<br>5_s_m1<br>1800_as_m1<br>4_as_m1<br>An2_as
3n_12<br>$T_{A}f$ = 58 °C<br>$T_{A}n$ = 58 °C	1985 – 2405	1500_s_m1<br>3200_as_m1	5_s_m1<br>ins_as	5_s_m1<br>ins_s_m1<br>ins_as
4n_12<br>$T_{A}f$ = 58 °C<br>$T_{A}n$ = 58 °C	2254 – 2994	5_s_m1<br>3800_as_m1	ins_s_m1<br>3200_as_m1	ins_s_m1<br>2600s<br>ins_as<br>3200_as_m1
B1<br>$T_{A}f$ = 58 °C<br>$T_{A}n$ = 58 °C	2750 – 3206	B_s<br>B/Cn1_as	Bn1_s<br>Bn1_as	Bn1_s<br>3150_s_m1<br>3200_as_m1

						Bn1_as
5n_12<br>T_Af = 68 °C<br>T_An = 68 °C	2986	–	4118	2600_s<br>9_as_m1	3150_s_m1<br>8_as	3150_s_m1<br>8_s<br>3800_as_m1<br>8_as
B2<br>T_Af = 58 °C<br>T_An = 68 °C	3419	–	3872	B_s<br>B/Cn1_as	C/Bn2_s<br>Bn2_as	C/Bn2_s<br>8_s<br>3800_as_m1<br>Bn2_as
C1<br>T_Af = 58 °C<br>T_An = 68 °C	3903	–	4340	C/Bn2_s<br>C_as	Cn1_s<br>B/Cn1_as	Cn1_s<br>9_s_m1<br>8_as<br>B/Cn1_as
6n_12<br>T_Af = 58 °C<br>T_An = 58 °C	4107	–	4628	8_s<br>10_as	9_s_m1<br>9_as_m1	9_s_m1<br>9_as_m1
C2<br>T_Af = 58 °C<br>T_An = 58 °C	4416	–	4873	C/Bn2_s<br>C_as	Cn2_s<br>Cn2_as	Cn2_s<br>10_s_m1<br>9_as_m1<br>Cn2_as
7n_12<br>T_Af = 58 °C<br>T_An = 58 °C	4604	–	5832	9_s_m1<br>12_as_m1	10_s_m1<br>11_as	10s_m1<br>11_s-neu<br>10_as<br>11_as
D1<br>T_Af = 58 °C<br>T_An = 68 °C	4983	–	5528	D_s<br>D/En1_as	Dn1_s<br>Dn1_as	Dn1_s<br>11_s-neu<br>10_as<br>Dn1_as
6n_7<br>T_Af = 58 °C<br>T_An = 58 °C	5305	–	6333	10_s_m1<br>13_as_m1	11_s-neu<br>12_as_m1	11_s-neu<br>12_s<br>11_as<br>12_as_m1
D2<br>T_Af = 58 °C<br>T_An = 58 °C	5672	–	6126	D_s<br>D/En1_as	E/Dn2_s<br>Dn2_as	E/Dn2_s<br>12_s<br>11_as
E1<br>T_Af = 58 °C<br>T_An = 68 °C	6076	–	6532	E/Dn2_s<br>E_as	En1_s<br>D/En1_as	En1_s<br>13_s<br>12_as_m1<br>D/En1_as
8n_12<br>T_Af = 58 °C<br>T_An = 58 °C	6303	–	6840	12_s<br>14_as_m1	13_s<br>13_as_m1	13_s<br>13_as_m1
E2<br>T_Af = 58 °C	6621	–	7067	E/Dn2_s<br>E_as	En2_s<br>En2_as	En2_s<br>14_s_m1

T_{A}n = 68 °C				13_as_m1<br>En2_as
9n_12<br>T_{A}f = 58 °C<br>T_{A}n = 58 °C	6822 – 7212	13_s<br>14_as_m1	14_s_m1<br>14_as_m1	14_s_m1<br>14_as_m1
3'n<br>T_{A}f = 68 °C<br>T_{A}n = 68 °C	6723 – 7256	3'-GS1_s<br>3'-RACE_as<br>LUP<br>SUP	3'-GS1n_s<br>NUP	3'-GS1n_s<br>14_s_m1<br>13_as_m1<br>14_as_m1

Table S1. Summary of overlapping genome regions sequenced from subtype 3c strain 14-16753. First round PCR primers were added to the RT-mix which was completely introduced into the first round PCR-mix after transcription. Smart Primer were added to the smart cDNA-mix which was partly used in the first round PCR-mix after smart cDNA-synthesis. T_{A}f, annealing temperature of first round PCR; T_{A}n, annealing temperature of nested PCR.
