# Supplementary material for: Isolation of Subtype 3c, 3e and 3f-Like Hepatitis E Virus Strains Stably Replicating to High Viral Loads in an Optimized Cell Culture System
Source: Viruses. 2019 May 28;11(6):483. doi: 10.3390/v11060483 (PMC6632007; doi:10.3390/v11060483)
Supplement: Supplementary file 1 [file viruses-11-00483-s001.zip › viruses-499710 final supplementary/Table S2.pdf]

Sequenced genome segments of subtype 3e strain 14-22707

Sequence Segment	Location (on 14-16753 genome, nested Primer excluded)	Primer First round PCR	Nested PCR	Sequencing
5'n_2n<br>T_Af = 68 °C<br>T_An = 68 °C	-7 – 315	5'-RACE_s<br>5'-GS2n_as<br>LUP<br>SUP	NUP<br>5'-GS4_as	Sn_s<br>5'-GS4n_as
5'n_4<br>T_Af = 58 °C<br>T_An = 58 °C	-7 – 175	5'-RACE_s<br>5'-GS4_as<br>LUP<br>SUP	NUP<br>5'-GS4n_as	1_s<br>5'-GS4n_as
Sn<br>T_Af = 58 °C<br>T_An = 58 °C	132 – 565	S_s<br>S_as	Sn_s<br>Sn_as	Sn_s<br>2_s<br>1_as<br>Sn_as
2n_12<br>T_Af = 58 °C<br>T_An = 58 °C	473 – 1031	1_s<br>3_as_m1	2_s<br>2_as_m1	2_s<br>2_as_m1
A1<br>T_Af = 58 °C<br>T_An = 58 °C	750 – 1198	A_s<br>A_as	An1_s<br>An1_as	An1_s<br>An1_as
Fn11<br>T_Af = 58 °C<br>T_An = 58 °C	1127 – 2065	F1_s<br>F1_as	Fn1_s<br>Fn1_as	Fn1_s<br>Fun2_s<br>3_as_m1<br>Fun2_as<br>Fn1_as
Fn22<br>T_Af = 58 °C<br>T_An = 58 °C	1164 – 2082	F2_s<br>F2_as	Fn2_s<br>Fn2_as	Fn2_s<br>4_s<br>3_as_m1<br>Fun2_as<br>Fn2_as
3n_12<br>T_Af = 58 °C<br>T_An = 58 °C	1985 – 2405	1500_s_m1<br>3200_as_m1	5_s_m1<br>ins_as	ins_as
Hn11<br>T_Af = 58 °C<br>T_An = 58 °C	2040 – 2346	H1_s<br>H1_as	H1n_s<br>H1n_as	H1n_s<br>F2_as<br>H1n_as
Gn11<br>T_Af = 58 °C<br>T_An = 58 °C	2284 – 3939	G1_s<br>G1_as	G2/Gn1_s<br>Gn1_as	G2/Gn1_s<br>Gun2s<br>Gun2_as<br>Bn1_as<br>Gun3_as

				Gn1_as
Gn22<br>T_Af = 68 °C<br>T_An = 68 °C	2326 – 4075	G2/Gn1_s<br>G2_as	Gn2_s<br>Gn2_as	Gn2_s<br>Gun3_s<br>Gun2_as<br>Gun3_as<br>Gun4_as<br>Gn2_as
In11<br>T_Af = 58 °C<br>T_An = 58 °C	3484 – 4271	I1_s<br>I1_as	I1n_s<br>I1n_as	I1n_s<br>8_s<br>9_s_m1<br>Gun3_as<br>Gn1_as<br>Gn2_as<br>I1n_as
C1<br>T_Af = 58 °C<br>T_An = 68 °C	3903 – 4340	C/Bn2_s<br>C_as	Cn1_s<br>B/Cn1_as	B/Cn1_as
6n_12<br>T_Af = 58 °C<br>T_An = 58 °C	4107 – 4628	8_s<br>10_as	9_s_m1<br>9_as_m1	9_s_m1<br>9_as_m1
Jn11<br>T_Af = 58 °C<br>T_An = 58 °C	4509 – 4687	J1_s<br>J1_as	J1n_s<br>J1n_as	J1n_s<br>9_as_m1<br>J1n_as
7n_12<br>T_Af = 58 °C<br>T_An = 58 °C	4604 – 5832	9_s_m1<br>12_as_m1	10_s_m1<br>11_as	10_s_m1<br>Dn1_s<br>11_s-neu<br>10_as<br>11_as
6n_7<br>T_Af = 58 °C<br>T_An = 58 °C	5305 – 6333	10_s_m1<br>13_as_m1	11_s-neu<br>12_as_m1	11_s-neu<br>12_s<br>11_as<br>12_as_m1
Kn11<br>T_Af = 58 °C<br>T_An = 58 °C	5717 – 5923	K1_s<br>K1_as	K1n_s<br>K1n_as	K1n_s<br>12_s<br>11_as<br>K1n_as
8n_12<br>T_Af = 58 °C<br>T_An = 58 °C	6303 – 6840	12_s<br>14_as_m1	13_s<br>13_as_m1	13_s<br>13_as_m1
9n_12<br>T_Af = 58 °C<br>T_An = 58 °C	6822 – 7212	13_s<br>14_as_m1	14_s_m1<br>14_as_m1	14s_m1<br>14_as_m1
3'n_2<br>T_Af = 68 °C	6609 – 7256	3'-GS2_s<br>3'-RACE_as	3'-GSn2_s<br>NUP	3'-GSn2_s<br>3'-GS3_s

T_An = 68 °C		LUP<br>SUP		3'-GSn3_s
3'n<br>T_Af = 68 °C<br>T_An = 68 °C	6723 – 7256	3'-GS1_s<br>3'-RACE_as<br>LUP<br>SUP	3'-GS1n_s<br>NUP	14_s_m1<br>14_as_m1
3'n_3<br>T_Af = 68 °C<br>T_An = 68 °C	7079 – 7256	3'-GS3_s<br>3'-RACE_as<br>LUP<br>SUP	3'-GSn3_s<br>NUP	3'-GSn3_s<br>14_as_m1

Table S2. Summary of overlapping genome regions sequenced from subtype 3e strain 14-22707. First round PCR primers were added to the RT-mix which was introduced into the first round PCR-mix after transcription. Smart Primer were added to the smart cDNA-mix which was partly used in the first round PCR-mix after smart cDNA-synthesis. T_Af, annealing temperature of first round PCR; T_An, annealing temperature of nested PCR.
