# Supplementary material for: Isolation of Subtype 3c, 3e and 3f-Like Hepatitis E Virus Strains Stably Replicating to High Viral Loads in an Optimized Cell Culture System
Source: Viruses. 2019 May 28;11(6):483. doi: 10.3390/v11060483 (PMC6632007; doi:10.3390/v11060483)
Supplement: Supplementary file 1 [file viruses-11-00483-s001.zip › viruses-499710 final supplementary/Table S3.pdf]

Sequenced genome segments of subtype 3f-like strain 15-22016

Sequence Segment	Location (on 14-16753 genome, nested Primer excluded)	Primer		
		First round PCR	Nested PCR	Sequencing
5'n_5n<br>T_Af = 68 °C<br>T_An = 58 °C	-7 – 138	5'-RACE_s<br>5'-GS5_as<br>LUP<br>SUP	NUP<br>5'-GS5n_as	1_s<br>5'-GS5n_as
Sn<br>T_Af = 58 °C<br>T_An = 58 °C	132 – 565	S_s<br>S_as	Sn_s<br>Sn_as	1_as
427n<br>T_Af = 68 °C<br>T_An = 58 °C	445 – 852	409_s<br>897_as	427_s<br>874_as	427_s<br>560g_s<br>An1_s<br>874_as
A1<br>T_Af = 58 °C<br>T_An = 68 °C	750 – 1198	A_s<br>A_as	An1_s<br>An1_as	An1_s<br>3_s<br>2_as_m1<br>An1_as
902n<br>T_Af = 58 °C<br>T_An = 58 °C	920 – 1805	560g_s<br>3034g_as	902g_s<br>1825g_as	902g_s<br>3_s<br>1542g_s<br>3_as_m1<br>1825g_as
1542n<br>T_Af = 68 °C<br>T_An = 68 °C	1560 – 2521	560g_s<br>3034g_as	1542g_s<br>2539g_as	1542g_s<br>1825g_as<br>2539g_as
2001n<br>T_Af = 58 °C<br>T_An = 58 °C	2019 – 2968	560g_s<br>3034g_as	2001g_s<br>2986g_as	2001g_s<br>2418g_s<br>2757g_s<br>2539g_as
2418n<br>T_Af = 68 °C<br>T_An = 68 °C	2438 – 3014	2001g_s<br>4276g_as	2418g_s<br>3034g_as	2418g_s<br>2757g_s<br>2539g_as<br>2986g_as<br>3034g_as
2757n<br>T_Af = 58 °C<br>T_An = 68 °C	2778 – 3682	2001g_s<br>4276g_as	2757g_s<br>3700g_as	2757g_s<br>3150_s_m1<br>3553g_s<br>2986g_as<br>3034g_as<br>3700g_as
3553n	3571 – 4121	2001g_s	3553g_s	3553g_s

T_Af = 68 °C<br>T_An = 68 °C		4276g_as	4143g_as	8_as<br>4143g_as
C1<br>T_Af = 58 °C<br>T_An = 68 °C	3903 – 4340	C/Bn2_s<br>C_as	Cn1_s<br>B/Cn1_as	Cn1_s<br>9_s_m1<br>B/Cn1_as
6n_12<br>T_Af = 58 °C<br>T_An = 58 °C	4107 – 4628	8_s<br>10_as	9_s_m1<br>9_as_m1	9_s_m1<br>B/Cn1_as<br>9_as_m1
7n_12<br>T_Af = 58 °C<br>T_An = 58 °C	4604 – 5832	9_s_m1<br>12_as_m1	10_s_m1<br>11_as	10s_m1<br>D_s<br>Dn1_s<br>11_s-neu<br>C_as<br>10_as<br>11_as
6n_7<br>T_Af = 58 °C<br>T_An = 58 °C	5305 – 6333	10_s_m1<br>13_as_m1	11_s-neu<br>12_as_m1	11_s-neu<br>12_as_m1
6196n<br>T_Af = 58 °C<br>T_An = 58 °C	6217 – 6367	6062_s<br>6525_as	6196_s<br>6387_as	6196_s<br>12_as_m1<br>6387_as
8n_12<br>T_Af = 58 °C<br>T_An = 58 °C	6303 – 6840	12_s<br>14_as_m1	13_s<br>13_as_m1	13_s<br>13_as_m1
3'n_4<br>T_Af = 58 °C<br>T_An = 58 °C	6756 – 7256	3'-GS4_s<br>3'-RACE_as<br>LUP<br>SUP	3'-GSn4_s<br>NUP	3'-GSn4s<br>3'-GS3_s<br>3'-GSn3_s<br>14_as_m1

Table S3. Summary of overlapping genome regions sequenced from subtype 3f-like strain 15-22016. First round PCR primers were added to the RT-mix which was introduced into the first round PCR-mix after transcription. Smart Primer were added to the smart cDNA-mix which was partly used in the first round PCR-mix after smart cDNA-synthesis. T_Af, annealing temperature of first round PCR; T_An, annealing temperature of nested PCR.
