# Supplementary material for: Isolation of Subtype 3c, 3e and 3f-Like Hepatitis E Virus Strains Stably Replicating to High Viral Loads in an Optimized Cell Culture System
Source: Viruses. 2019 May 28;11(6):483. doi: 10.3390/v11060483 (PMC6632007; doi:10.3390/v11060483)
Supplement: Supplementary file 1 [file viruses-11-00483-s001.zip › viruses-499710 final supplementary/Table S4.pdf]

Primers used for whole genome sequencing

	Primer	Sequence (5' – 3')	Binding position (on 14-16753 genome)
‡	5'-RACE_s	AAGCAGTGGTATCAACGCAGAGTACGCGGG	on 5' $(dCTP)_n$ tail
†	1_s	GCAGACCACGTATGTGGTCGA	2 – 22
	S_s	CGGCCAATTCTGCCCTGG	94 – 111
	Sn_s	CGAATGCTGTGGTGGTTCGG	112 – 131
§	5'-GS5n_as	TCTCAGTTTGTACACGGGACA	139 – 159
§	5'-GS5_as	GCTGTCGGGGTTGCATCAGGTTAATA	164 – 189
§	5'-GS4n_as	GAAATACGAGCTGCCGGGGTTGC	176 – 198
	289_as	TCTAGACAACGACCAGCCCG	270 – 289
§	5'-GS4_as	CACCGGTGCAAGACATTAGGGTTATCAT	316 – 343
§	5'-GS2n_as	GGGTCGGGGCAGAATACCAACGCTGT	371 – 396
	393_as	TAGGAGCAGAGTACCAGCGC	374 – 393
	409_s	CTAACTGCCGCCGTTCTG	409 – 426
	440_as	GAGGCCACGCAAAGCAGAAC	421 – 440
	427_s	CACTGCGTGGTTTGCCCC	427 – 444
†	2_s	GATCGTACCTAYTGYTTTGATGG	450 – 472
§	5'-GS1n_as	CCCCAGTTTCTGCGGCAAATGAGC	484 – 507
§	5'-GS1_as	CCGGCCAGAGGTCATGCAGAGAATAAAG	513 – 540
†	1_as	GGGCCATAGCCTCBGCAACATC	543 – 564
	560g_s	GGCCCGYCAYGGGATGAC	560 – 577
	Sn_as	CAAGCGTGTCATCCCGTGC	566 – 584
	A_s	CGGTGATCGTGCTGTTGTGAC	665 – 685
	S_as	GGTCACAACAGCACGATCACC	666 – 686
	An1_s	CATACTTCGTGCATGGATCCGC	728 – 749
	874_as	CGAGGGTACGGAACATAAGGCA	853 – 874
	897_as	ACCTAACATACACCTCCGTTGA	876 – 897
	902g_s	ATTYGGCCCTGGYGGGTC	902 – 919
	F1_s	TCATGCTGTTCCGGTCCACA	962 – 981
†	3_s	CATATTTGGGACCGGCTYATGCT	978 – 1000
†	2_as_m1	TAGGTCATAAGYCGTGARCAGCA	1032 – 1054
	F2_s	TCCGCGGGATCAGTTATAAGGT	1057 – 1078
	Fn1_s	GGTTGGAACGCATCAGAGGA	1107 – 1126
	Fn2_s	TGATTACCGCGGCGTATTTAACT	1141 – 1163
	An1_as	GTCGGCGCATACCCTTGGAT	1199 – 1218
	Fun2_s	CAGTGCCGCCGCTGGCT	1323 – 1339
†	4_s	GGGTAAGTTCTGCTGYTTTATG	1427 – 1448
†	3_as_m1	TCYAAAAAGCAGGTRCACTCCTG	1461 – 1483
†	1500_s_m1	CTGCTTTTTRGAACCAGCAGAGG	1472 – 1494
	1542g_s	GARGTGGACCCYGCTGAG	1542 – 1559
	An2_s	TGACTGCCACCGTCGAACTC	1669 – 1688
†	1800_as_m1	KGACTGGCGAGAGGCRTCRAAC	1805 – 1826

	1825g_as	GCCTGGCGRGARTCATCAAA	1806 – 1825
	Fun2_as	CAGGGGTGAGCTCATATG	1852 – 1869
	H1_s	AGACTGTACTGCTGTTTTCCCC	1904 – 1925
†	5_s_m1	GCAGCCTTTTGCAGTGCYCTTTA	1962 – 1984
	2001g_s	ACCCAGCGYCATTCGCTG	2001 – 2018
	H1n_s	GGTGGGCTGTGGTTGCAC	2022 – 2039
†	4_as_m1	GGGAARATACCCAACARCCCCTC	2043 – 2065
	Fn1_as	GTGTGCCCGGGAGAAAAGG	2066 – 2084
	Fn2_as	TTTGCGGACTCCCAGGTGT	2083 – 2101
	An2_as	CATGTCCGGGTGTACAAAGTTCC	2118 – 2140
	F1_as	CCAGGTCCGGGTGTAAAGTGT	2121 – 2141
	A_as	CAGGAGGTGTAGAGTGGCGT	2216 – 2235
†	ins_s_m1	CCTGTTAGTGACRTTTGGGTGT	2232 – 2253
	F2_as	GCACCCAAGCGTCACTAACA	2234 – 2253
	G1_s	TAGTGACGCTTGGGTGCTTC	2237 – 2256
	G2/Gn1_s	TCTGAGGGGTCGGTGGCTA	2265 – 2283
	Gn2_s	TAGTAAGCCCACTAGCCCGC	2306 – 2325
	H1n_as	TGGTGGCTTACGAACAGGGG	2347 – 2366
†	2600_s	ACCTATCCGGATGGGGCAAAGG	2403 – 2424
†	ins_as	ACACCTTTGCCCCATCCGGATA	2406 – 2427
	2418g_s	GCYAAGGTGTAYGCAGGCTC	2418 – 2437
	H1_as	AGGGTTGGACGCATTGACC	2465 – 2483
	2539g_as	GACTCCGGRAAVCGCTGG	2522 – 2539
	Gun2_s	CCGATCATTCATGCTGTGG	2601 – 2619
	Gun3_s	GCGTACCGTGAGACCTG	2664 – 2680
	Gun2_as	AGTGGGTAAGCGGCTGTG	2693 – 2710
	B_s	CTACCCACTCCTCGGCTC	2702 – 2719
	Bn1_s	ACCAAGTTCCCGTTAGCCTCAG	2728 – 2749
	2757g_s	GCCTGGGARCGTAACCAYCGC	2757 – 2777
†	3150_s_m1	TATCAGTTYACTGCTGGGGTCC	2964 – 2985
	2986g_as	GGGACCCCGGCRGTRAAC	2969 – 2986
†	3200_as_m1	TGCTGTATAGARCGCGACTTCC	2995 – 3016
	3034g_as	ACAACRTCAACATCSCCCTG	3015 – 3034
	Bn1_as	TGGTTTGGGTCGCCGAGAAG	3207 – 3226
	I1_s	TTCAGACTACTAGCCGCGTT	3364 – 3383
	C/Bn2_s	TCTGGAATGAGCCTGCCATTGG	3397 – 3418
	I1n_s	CGCAATCACCGTCCATGAGG	3464 – 3483
	3553g_s	CYCGYGCACATGCCATCG	3553 – 3570
	Gun3_as	ACACATTTCTCTGTGTGGC	3583 – 3601
†	8_s	CCCCCGGCTTGTTWCGTGAGGT	3613 – 3634
	3800_as_m1	ACCRACCTCACGTAACAAGCCG	3617 – 3638
	3700g_as	GGGCGRTGRTGGCCCACT	3683 – 3700
	Bn2_as	ACCGTGAGCTCCTGAGGCAT	3873 – 3892
	Cn1_s	AGCTCACGGTGTCTGATAGC	3883 – 3902

	Gun4_as	TGTCCGTGAGCTCAAAAACC	3908	–	3927
	Gn1_as	CTGACTAGGGGCCGCCATTC	3940	–	3959
	G1_as	CCTCATACAACTTCGTCCGGC	4000	–	4020
	Gn2_as	ACATGTGGTGGCCTGGACC	4076	–	4094
†	9_s_m1	CYACYACGTGTGAGTTRTATGAG	4083	–	4106
†	8_as	CCATCCTGACCTTTCTCCACCAT	4119	–	4141
	G2_as	CGTCCTGGCCTTTCTCAACC	4121	–	4140
	4143g_as	AGCCRTCCTGRCCCTTCTCCAC	4122	–	4143
	4276g_as	CTCCARGCCGARATGCCC	4259	–	4276
	I1n_as	GGGCACAGAAGGTCTTACTCCA	4272	–	4293
	I1_as	AGGTGGGAGTAGGGCTAAGA	4330	–	4349
	B/Cn1_as	CCGTAGAAAATATTAGGCGGGAG	4341	–	4363
	Cn2_s	CTGTCTCAGGGGCAGGTTCA	4396	–	4415
	J1_s	AGCCGGCTCTAGTATGGTCT	4406	–	4425
	J1n_s	GCGTTATCATGGAAGAGTGTGGC	4486	–	4508
†	10_s_m1	CTYAAGGGATTTTGGAAGAARCA	4581	–	4603
†	9_as_m1	GCCATRTTCCARACAGTRTTCCA	4629	–	4651
	J1n_as	TCCCCTTTGAACGCTGCTACT	4688	–	4708
	J1_as	CCACAACCAGCAATCAAGGC	4758	–	4777
	Cn2_as	TTCTCAGACAGCCGGCCG	4874	–	4891
	D_s	AGAAGAACTGGGGCCCTGGG	4888	–	4907
	C_as	CACATACCTGCGCAACATTCGT	4962	–	4983
	Dn1_s	ACGAATGTTGCGCAGGTATGT	4962	–	4982
†	11_s-neu	CGGCAGTGGTTTCTGGRGTGAC	5283	–	5304
†	10_as	GCGAAGGGCTGAGAATCAACCC	5306	–	5327
	Dn1_as	TCAACATCAGGTACAGGGGCT	5529	–	5549
	K1_s	TCTATATGCCGCCCCGCTC	5634	–	5652
	E/Dn2_s	AAACCCTCTGCTGCCCCTTC	5652	–	5671
	K1n_s	GGCAACTGAGGCGTCTAATT	5697	–	5716
†	12_s	CTAATTATGCCCAGTAYCGGGTTG	5711	–	5734
†	11_as	GTAATAGAGTTCATRTCAACAGA	5833	–	5855
	K1n_as	ACCCTGATTGCGGTAATGGA	5924	–	5943
	K1_as	AGGGGAGCCATGAATACAAAGC	6003	–	6024
	En1_s	GGGCGCTTGGACTCCTTGAT	6056	–	6075
	6062_s	TGGGGCTTCTCGATTTTGCAC	6062	–	6082
	Dn2_as	TGCTTGTATACCGGGACACACG	6127	–	6148
	6196_s	ACAACTACTGCAGCCACACGT	6196	–	6216
†	13_s	CTCACACTCTTTAATCTTGCTGA	6280	–	6302
†	12_as_m1	AACTGTCCCCCRGCCGACGAAAT	6334	–	6356
§	3'-GS2_s	TCCCGCCCAGTTGTCTCAGCCAATGG	6364	–	6389
	6387_as	ATTGGCTGAGACGACTGGGC	6368	–	6387
	6525_as	CCGATCCTGTTCATGCTGGTTG	6504	–	6525
§	3'-GS1_s	ACCCCTTCGCCTGCCCCTTCTCG	6529	–	6551
	D/En1_as	CGAGAAGGGGCAGGCGAAG	6533	–	6551

§	3'-GSn2_s	TTGTGGCTCTCTCTCACTGCTGCCGA	6583 – 6608
	En2_s	GCCGCCGAGTATGACCAGAC	6601 – 6620
§	3'-GS4_s	TGTTAATGTGGCGACGGGTGCTCAGG	6672 – 6697
§	3'-GS1n_s	GCTGTCGCCCGTTCCCTTGACTGGTC	6697 – 6722
§	3'-GSn4_s	GACGGTCGGCCTCTCACTAC	6736 – 6755
†	14_s_m1	GGAAGTTATCYTTTTGGGAGGC	6800 – 6821
§	3'-GS3_s	TTTTGGGAGGCTGGCACTACTAAGGC	6811 – 6836
†	13_as_m1	AGTTGTGTTRTAATTATASGGGTA	6841 – 6864
§	3'-GSn3_s	CCCTGAGTGCCGTAATCTTGGCCTAC	7053 – 7078
	En2_as	CACACCCCTGCAAACCGAGA	7068 – 7087
	E_as	TCAGCAATAGTAGACTGGAAGGC	7087 – 7109
†	14_as_m1	TTTTTTTTTTTTCCWGGGAGCGCG	7213 – 7236
‡	3'-RACE_as	AAGCAGTGGTATCAACGCAGAGTAC$(T)_{30}$VN	on 3' poly(A) tail
‡	LUP	CTAATACGACTCACTATAGGGCAAGCAGTGGTATCAACGCAGAGT	on 5'-RACEs or 3'-RACEas
‡	SUP	CTAATACGACTCACTATAGGGC	on LUP
‡	NUP	AAGCAGTGGTATCAACGCAGAGT	on LUP

Table S4. Positions and nucleotide sequences of primers used for whole genome sequencing.
† Primers based on Johne et al. [1]; m1, modified; s, sense; as, antisense; ‡ primers based on BD SMART™ RACE cDNA Amplification Kit User Manual; § primers designed according to recommendations of BD SMART™ RACE cDNA Amplification Kit User Manual.

Supplementary Reference

1. Johne R, Reetz J, Ulrich RG, et al. An ORF1-rearranged hepatitis E virus derived from a chronically infected patient efficiently replicates in cell culture. *Journal of Viral Hepatitis*. 2014;21(6):447-456. doi:10.1111/jvh.12157.
